# Gastrointestinal and genitourinary toxicity profiles of metformin versus placebo in men with prostate cancer receiving prostate radiotherapy: interim toxicity results of a double-blinded, multicenter, phase II randomized controlled trial

**DOI:** 10.1186/s13014-021-01935-x

**Published:** 2021-11-04

**Authors:** Julian O. Kim, Megan O. McDonald, Aldrich Ong, Rashmi Koul, Arbind Dubey, William Hunter, Shahida Ahmed, Harvey Quon, Don Yee, Matthew Parliament, Gokulan Sivananthan, Brita Danielson, Lindsay Rowe, Sunita Ghosh, Nawaid Usmani

**Affiliations:** 1 Radiation Oncology, Department of Radiology, Max Rady Faculty of Medicine, University of Manitoba, Winnipeg, Manitoba Canada; 2 CancerCare Manitoba Research Institute, CancerCare Manitoba, 675 McDermot Ave, Winnipeg, Manitoba R3E 0V9 Canada; 3 Postgraduate Medical Education, Max Rady Faculty of Medicine, University of Manitoba, Winnipeg, Manitoba Canada; 4 Western Manitoba Cancer Center, Brandon, Manitoba Canada; 5 Division of Radiation Oncology, Department of Oncology, University of Calgary, Calgary, Alberta Canada; 6 Division of Radiation Oncology, Department of Oncology, University of Alberta, Edmonton, Alberta Canada; 7 Department of Oncology, University of Alberta, Edmonton, Alberta Canada

**Keywords:** Metformin, Prostate cancer, Gastrointestinal toxicity, Genitourinary toxicity

## Abstract

Androgen deprivation therapy (ADT) used for prostate cancer (PCa) management is associated with metabolic and anthropometric toxicity. Metformin given concurrent to ADT is hypothesized to counteract these changes. This planned interim analysis reports the gastrointestinal and genitourinary toxicity profiles of PCa patients receiving ADT and prostate/pelvic radiotherapy plus metformin versus placebo as part of a phase 2 randomized controlled trial. Men with intermediate or high-risk PCa were randomized 1:1 to metformin versus placebo. Both groups were given ADT for 18–36 months with minimum 2-month neoadjuvant phase prior to radiotherapy. Acute gastrointestinal and genitourinary toxicities were quantified using CTCAE v4.0. Differences in ≥ grade 2 toxicities by treatment were assessed by chi-squared test. 83 patients were enrolled with 44 patients randomized to placebo and 39 randomized to metformin. There were no significant differences at any time point in ≥ grade 2 gastrointestinal toxicities or overall gastrointestinal toxicity. Overall ≥ grade 2 gastrointestinal toxicity was low prior to radiotherapy (7.9% (placebo) vs. 3.1% (metformin), *p* = 0.39) and at the end of radiotherapy (2.8% (placebo) vs 3.1% (metformin), *p* = 0.64). There were no differences in overall ≥ grade 2 genitourinary toxicity between treatment arms (19.0% (placebo) vs. 9.4% (metformin), *p* = 0.30). Metformin added to radiotherapy and ADT did not increase rates of ≥ grade 2 gastrointestinal or genitourinary toxicity and is generally safe and well-tolerated.

**Supplementary Information:**

The online version contains supplementary material available at 10.1186/s13014-021-01935-x.

## Background

Androgen deprivation therapy (ADT), a cornerstone of modern prostate cancer (PCa) management [1], is associated with improved survival amongst men with high-risk prostate cancer (PCa) when added to radiotherapy [2–5]. However, ADT induced hypogonadism is associated with metabolic derangements (dyslipidemia [6], hyperinsulinemia [7, 8], and insulin resistance [6–8]) and anthropometric changes (weight gain, centralized obesity [9, 10]) which can lead to metabolic syndrome, diabetes, and cardiovascular disease [11].

Metformin, an economical anti-hyperglycemic medication, is known to decrease/stabilize weight, decrease plasma triglycerides, and reduce diabetes incidence and complications [12, 13]. There is considerable interest in employing adjunctive metformin to potentially counteract the metabolic and anthropometric changes associated with ADT, and potentially improve PCa outcomes. [14, 15].

Metformin is associated with mild/moderate gastrointestinal (GI) side effects that ameliorate with dose titration [12, 13]. Approximately 20% of patients will experience diarrhea, abdominal discomfort, anorexia, nausea, or bloating during metformin initiation [12, 13]. The toxicity of metformin concurrent to prostate/pelvic radiotherapy and ADT is unknown. This planned interim analysis of the PREMIUM trial (Prevention of Metabolic Syndrome and Increased Weight Using Metformin Concurrent to Androgen Deprivation Therapy for Locally Advanced Adenocarcinoma of the prostate, Clinicaltrials.gov trial identifier NCT01996696), reports the gastrointestinal and genitourinary toxicity profiles of high-risk PCa patients undergoing ADT and prostate/pelvic radiotherapy plus metformin versus placebo on a phase 2 randomized controlled trial (RCT).

## Methods and Materials

### Patient selection

Eligible patients were males ≥ 18 years old, Eastern Cooperative Oncology Group (ECOG) 0 to 1 and normoglycemic (Fasting Plasma Glucose ≤ 6.9 mmol/L or HemoglobinA1C (HgbA1C) < 6.5%) with biopsy confirmed High- Tier Intermediate risk (≥ 2 of: Gleason Score (GS) = 7, PSA of 10–20 ng/mL, or ≥ 50% of biopsy cores containing GS 7) or high-risk PCa (any of: T3 disease, GS ≥ 8.0, and/or PSA ≥ 20 ng/mL) receiving curative intent ADT and external beam radiotherapy (EBRT). Patients with renal impairment (defined as eGFR < 45 mL/minute/1.73 m^2^) were excluded from trial. Patients at risk for lactic acidosis, including those with impaired renal function, liver disease including alcoholic liver disease, current alcohol abuse (≥ 3 alcoholic beverages per day), or severe infection were excluded from trial.

#### Study design and treatments

Participants were randomized (1:1), stratified by treatment center, to metformin 500 mg by mouth (PO) 3 times daily for 30 to 36 months or identical placebo. Metformin was titrated as follows: 500 mg by PO daily for two weeks, then 500 mg PO twice daily for two weeks, then 500 mg PO three times daily for the remainder of treatment. Study drugs were initiated 2 months (minimum) prior to the start of radiation. Both groups were given luteinizing hormone-releasing hormone (LHRH) agonist injections for 18–36 months with a minimum 2-month neoadjuvant phase prior to EBRT. RT consisted of elective pelvic nodal RT consisting of 46 Gy/23# or 50.4 Gy/28# (recommended by protocol) plus prostate boost RT to a total of 78 Gy/39# (or interstitial brachytherapy boost to 110–115 Gy or hypofractionated EBRT equivalent). Acceptable EBRT total prostate doses included: 70 Gy/28#, 68 Gy/25#, or 60 Gy/20#. All EBRT treatments utilized intensity modulated radiotherapy (IMRT) or volumetric modulated arc therapy (VMAT).

#### Statistical considerations

Planned sample size was 104 patients (97% power, 2-tailed α of 0.05 to detect a 4 kg difference in weight at 12 months). This pre-planned safety interim analysis was triggered after 52 patients completed 12 months follow-up to assess toxicity levels. Acute and subacute GI and GU toxicity was quantified using the Common Terminology Criteria for Adverse Events version 4.0 at: month 0 (baseline), month 3 (Pre-RT), month 5 (End of RT), and 12 months. Baseline characteristics were tabulated by treatment arm, and differences in characteristics were assessed using standard parametric and non-parametric tests. Differences in ≥ grade 2 toxicities were assessed by chi-squared test.

## Results

**Fig. 1:**
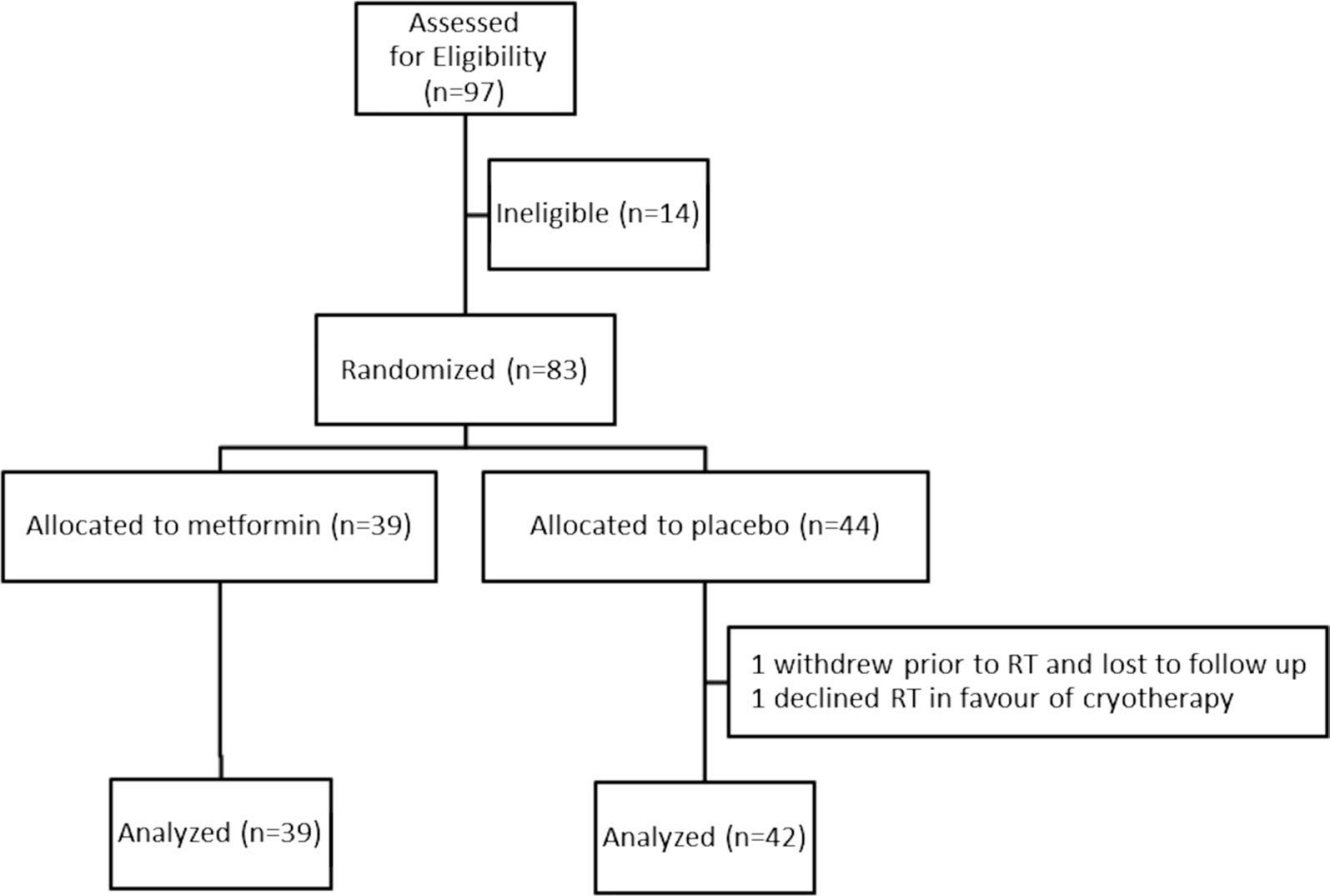
Enrollment and treatment allocation

At the time of interim analysis (frozen for analysis 15/01/2020) 83 patients were enrolled between December 2015 and September 2019 at three participating centers with mean follow-up of 27.3 months (range 0.5–63.2). Fourty-four patients were randomized to placebo and 39 were randomized to metformin. Two patients randomized to receive placebo did not receive radiotherapy; one of which withdrew from study prior to radiotherapy and was lost to follow-up, and the other patient declined radiotherapy in favour of cryotherapy. Eighty-one patients were included for analysis (Fig. 1).

Baseline characteristics of the cohort included: mean age of 72 years (SD 7.1; range 49–86), mean body mass index 30.3 kg/m^2^ (SD 5.5; range 22.2–52.5), median Gleason score 9 (range 7–9), and mean HgbA1C was 5.6% (range 4.9–6.4) (Table 1). All patients completed RT, with most receiving EBRT prostate boost total doses of 76–78 Gy/38–39# (62% (Metformin arm), 57% (placebo Arm)), or other hypofractionated schedules. Pelvic nodal radiation was used for most participants (71% (Metformin arm), 79% (placebo arm)). Interstitial brachytherapy boost was done in the minority (10% (Metformin arm), 12% (placebo arm)). One patient (metformin arm) had clinical node positive disease with a single left sided internal iliac lymph which was boosted to a total dose of 74 Gy in 2 Gy fractions. The remainder of patients were node negative. There were no statistically significant differences in baseline patient, disease, or treatment characteristics by arm.

**Table 1:** Baseline characteristics of the patients

Characteristic*	Metformin (n = 39)	Placebo (n = 42)	*p* value
Age	71 (56–82)	73 (49–86)	0.22
Weight (kg)	95.5 (62.7–157.0)	91.6 (71.8–126.1)	0.24
Waist Circumference (cm)	110 (81.5–185)	109 (92–185)	0.60
BMI (kg/m^2^)	30.3 (22.2–52.5)	29.8 (23.7–40.4)	0.40
Mean SBP (mmHg)	145 (108–179)	142.5 (103–173.5)	0.92
HbA1C (%)	5.6 (5.1–6.4)	5.6 (4.9–6.4)	0.95
Smoking Pack-Year-History	15 (0–107.5)	20 (0–75)	0.56
Marital Status—*no. (%) married*	31 (79.5)	37 (88.1)	0.29
ECOG	0 (0–1)	0 (0–1)	0.59
Total IPSS	11 (0–31)	10 (0–22)	0.86
Gleason Score	9 (7–9)	8 (7–9)	0.44
% Biopsy Cores Positive	7 (2–14)	7 (2–12)	0.62
Clinical T-Stage—*no. (%)* T1 T2 T3	13 (33) 16 (41) 10 (26)	13 (31) 16 (38) 13 (31)	0.87
Pelvic Nodal Irradiation—*no. (%)*	30 (71)	33 (79)	0.82
Prostate Boost Type—*no. (%)*			
Standard Fractionation (76/38 to 78/39)	﻿24 (62)	24 (57)	0.90
Hypofractionated (60/20, 70/28, 72.8/28)	11 (28)	13 (31)	
Interstitial Brachytherapy Boost	4 (10)	5 (12)	

^*^Values given as mean (range) unless otherwise indicated

Abbreviations: SBP = Systolic Blood Pressure; HbA1c = Hemoglobin A1C; ECOG = Eastern Cooperative Oncology Group; IPSS = International Prostate Symptom Score

Six patients randomized to receive metformin and 12 patients randomized to receive placebo discontinued the study drug prior to 12 months follow-up. Two patients discontinued early due to gastrointestinal side effects including: one patient from the placebo arm who discontinued at 4 months (during RT) due to grade 1 diarrhea, and one patient from the metformin arm who discontinued at 6 months (post-RT) due grade 1 bloating. All other patients who discontinued study drug did so due to patient preference. No patients discontinued the study due to ≥ grade 3 GI or GU toxicity.

**Fig. 2:**
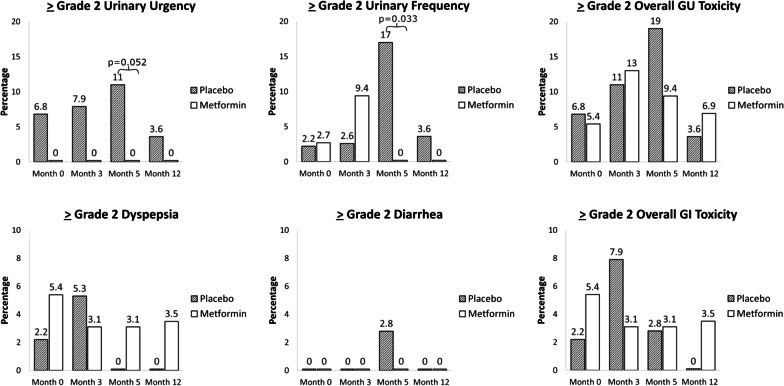
Genitourinary and gastrointestinal toxicity by treatment

Amongst the 81 participants analyzed, there were no significant difference in ≥ grade 2 GI toxicities including bloating, dyspepsia, nausea, diarrhea or overall GI toxicity at any time during follow-up (Fig. 2, Supplemental Table 1). Overall ≥ grade 2 GI toxicity was low at 3 months follow-up prior to RT (7.9% (placebo) vs. 3.1% Metformin), *p* = 0.39) and at 5 months follow-up at the end of RT (2.8% (placebo) vs 3.1% (Metformin), *p* = 0.64). Patients receiving metformin experienced no ≥ grade 2 urinary urgency during RT (11.1% (placebo) vs 0% (metformin), *p* = 0.052). Those receiving metformin had no increased urinary frequency during RT (16.7% (placebo) vs. 0% (metformin), p = 0.033). There were no differences in overall ≥ grade 2 GU toxicity between arms (19.0% (placebo) vs. 9.4% (metformin), *p* = 0.30). There were no ≥ grade 3 overall GI, GU or ADT-associated toxicities reported during follow-up. No patients developed lactic acidosis during follow-up.

## Discussion

Metformin has been postulated to stabilize or prevent some of the adverse metabolic effects of ADT. However, there is paucity of data regarding the toxicity profile of the combination of metformin concurrent to pelvic/prostate radiotherapy. In this study, we did not detect any evidence that metformin increased acute or subacute gastrointestinal or genitourinary toxicity.

Furthermore, toxicity rates reported herein align with previously reported acute GI and GU toxicities of prostate radiotherapy using modern VMAT or IMRT techniques [16–18]. Although data is limited due to the relatively recent introduction of VMAT or IMRT, ≥ grade 2 acute GI toxicities are reported at rates of 2.3- 4% and ≥ grade 2 acute GU toxicities are reported at rates of 7- 8.5% [16, 17]. Overall combined ≥ grade 2 acute GI and GU toxicities combined rates reported were 9.7% [16].

These interim results detected no increases in GI or GU toxicity with metformin added to ADT and pelvic radiotherapy. The titration period of metformin, which coincided with the neoadjuvant phase of ADT, likely provided adequate time for participants to adjust to GI side effects of metformin prior to the start of radiotherapy.

## Conclusions

Metformin added to radiotherapy and ADT did not increase rates of ≥ grade 2 GI or GU toxicity. While these findings are preliminary, the addition of metformin to RT and ADT appears to be safe and well-tolerated.

## Supplementary Information

**Additional file 1**. Table S1: Gastrointestinal and genitourinary toxicities by treatment.

## Data Availability

The datasets generated and/or analyzed during the current study are not publicly available due to it being an interim analysis with ongoing data collection for the study, but will be available in the future following final analysis from the corresponding author on reasonable request.

## Abbreviations

ADT: Androgen deprivation therapy
PCa: Prostate cancer
GI: Gastrointestinal
RCT: Randomized controlled trial
ECOG: Eastern Cooperative Oncology Group
HgbA1c: Hemoglobin A1c
GS: Gleason score
EBRT: External beam radiotherapy
LHRH: Luteinizing hormone-releasing hormone
IMRT: Intensity modulated radiotherapy
VMAT: Volumetric modulated arc therapy
CTCAE: Common Terminology Criteria for Adverse Events
